# Programmable Transition between Adhesive/Anti-Adhesive Performances on Porous PVDF Spheres Supported by Shape Memory PLLA

**DOI:** 10.3390/polym14030374

**Published:** 2022-01-19

**Authors:** Jiaqin Zhao, Liang Zhang, Xiong Cheng, Jiayao Wang, Yongjin Li, Jichun You

**Affiliations:** College of Material, Chemistry and Chemical Engineering, Hangzhou Normal University, Hangzhou 311121, China; Jiaqin_Zhao@163.com (J.Z.); liangzhang1128@gmail.com (L.Z.); 2019112009005@hznu.stu.edu.cn (X.C.); jiayao-wang@outlook.com (J.W.); yongjin_li@hznu.edu.cn (Y.L.)

**Keywords:** adhesive/anti-adhesive performances, shape memory effect, porous spheres, PLLA, PVDF

## Abstract

Superhydrophobic surfaces with switchable adhesive/anti-adhesive performances are highly desired but still challenging. Herein, by loading porous poly (vinylidene fluoride) (PVDF) spheres on a shape memory polylactic acid (PLLA) film, a quasi-superhydrophobic surface of composite film (PVDF@PLLA) with the ability to tailor its surface structures/composition and related adhesive behaviors was fabricated. The as-prepared surface is covered by porous PVDF spheres. The combination of hydrophobicity of PVDF and hierarchical roughness resulted from porous spheres contributing to the high contact angle and low sliding angle, corresponding to Cassie state and lotus leaves effect. Upon uniaxial or biaxial tension, the distance among hydrophobic spheres is so high that more and more hydrophilic defects (PLLA film) have been exposed to water droplets, accounting for the quasi-superhydrophobic surface with a higher sliding angle. This is the reason for the Wenzel state and rose petals effect. After heating, PLLA film recovers to its original state. The porous PVDF spheres cover the whole film again, leading to the enhanced mobility of water droplets on the surface. The transition between the rose petals effect and the lotus leaves effect is programmable and reversible. Our result provides a novel strategy to tailor adhesive behaviors by combining (quasi-)superhydrophobic surface with shape memory effect.

## 1. Introduction

In nature, many plants and insects, including plant leaves, the wing of the butterfly and the leg of the water strider, exhibit excellent superhydrophobicity [1,2,3,4]. A superhydrophobic surface, defined as the one with a water contact angle larger than 150°, has been widely used in various applications [5,6,7,8,9,10,11,12]. Both static water contact angle (CA) and sliding angle (SA) have been employed to assess the performance of superhydrophobic surfaces. The former corresponds to the wettability of certain liquids while the latter represents the contact angle hysteresis. In the past few years, two typical superhydrophobic examples of lotus leaves and rose petals have been paid much attention because of their interesting properties [13,14,15,16,17,18,19,20,21,22]. Water droplets on the former exhibit high CA and low SA, while high CA and high SA can be observed on the latter. In other words, lotus leaves and rose petals correspond to anti-adhesive and adhesive surfaces, respectively, which play key roles in many fields. For instance, the combination of them endows the surface containing micro-arrays with a special ability in achieving programmable droplet motion and transportation along a certain direction [21]. The transition between them, therefore, is highly desired but still challenging.

It is well known that wettability is under the control of surface composition and roughness. To achieve the transition between adhesion and anti-adhesion, much effort has been made according to the manipulation strategy of chemical composition and micro-structures on the target surface, as well as the combination of them. Firstly, it is possible to achieve this transition by varying chemical composition [18,19]. Su and his co-workers introduced hydrophilic defects on polyelectrolyte multilayer (PEM) surfaces supported by rough silica nanoparticles. These hydrophilic regions lead to the obvious collapse of water droplets into gaps among neighboring particles. This is the reason for the transition from an anti-adhesive state to an adhesive state [18]. Secondly, roughness plays an important role in the transition [20,21]. Chen et al. realized the transition between lotus leaves and rose petals effect by manipulating pillar array [21]. Upon stretching, the period of micro/nanostructure on the homogeneous surface of the film increased remarkably, accounting for the lower magnitude of surface roughness and the resultant transition from pinning to rolling performance. Finally, simultaneous adjustment of the chemical composition and surface geometry can act as an efficient method for this transition. In our previous work, thermoplastic polyurethane electrospun fibers have been used as the bottom layer to fabricate composite fabrics of TPU/MWCNTs (multi-walled carbon nanotubes) [22]. The attained fabrics exhibit high CA and low SA due to the existence of MWCNTs on their surface, corresponding to the lotus leaves effect. Uniaxial tension produced reduced surface roughness and complicated chemical composition of the surface, contributing to the rose petals effect. 

**Scheme 1:**
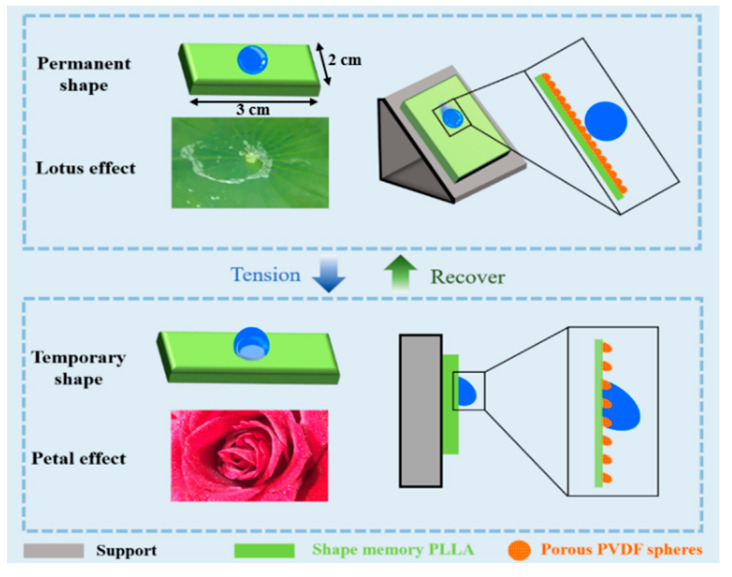
Schematic diagram of droplet anti-adhesion/adhesion mechanism on the PVDF@PLLA composite surface.

So far, some strategies for the transition discussed above have become available. In the efficient rolling-pinning transition on a superhydrophobic surface with controllable adhesive performance, however, there are still some open problems. On one hand, programmable transition, which means that the adhesive/anti-adhesive states can be fixed in some cases while the transition can be triggered when required, remains challenging. On the other hand, the developed strategy concerns either the requirement of special equipment (e.g., a micro-array template in [20]) or high cost (MWCNTs in [22]), which does limit its mass production and applications. A facile strategy for fabrication and transition has been highly desired. In this work, therefore, it is proposed to achieve the programmable transition according to the idea shown in Scheme 1, in which the shape memory PLLA films are covered by porous PVDF spheres [23]. After preparation, the surface of PVDF@PLLA composite film exhibits the lotus leaves effect because of the hydrophobic performance and roughness resulting from neighboring spheres, corresponding to a high contact angle and low sliding angle. Upon uniaxial or biaxial tension, there is obvious deformation on PLLA films, leading to the exposed area which has not been covered by porous PVDF spheres. This is the reason for the occurrence of hydrophilic defects (PLLA) on hydrophobic PVDF surfaces, accounting for the enhanced contact angle hysteresis, i.e., rose petals effect. The transition between them is programmable, reversible and easy to handle. It can be well controlled based on shape fix and the recovery abilities of the shape memory effect of PLLA. 

## 2. Experimental Section

### 2.1. Materials

Polymethyl methacrylate (PMMA, M_w_ = 15,000 g/mol, PDI = 1.58) and PLLA (3001D, M_n_ = 89,300 g/mol, PDI = 1.80) were purchased from Aldrich (Shanghai, China) and Nature Works (Blair, NE, USA), respectively. PVDF samples were provided by Scientific Polymer Products Ins. The molecular weight and polydispersity of the PVDF were: M_w_ = 209,000 g/mol and PDI = 2.0, respectively. P(MMA-co-GMA) (SZ-01, M_n_ = 40,000 g/mol, PDI = 2.0, the structural formula is shown in Figure S1) containing 8 wt% GMA monomer units were purchased from Aikechem (Hangzhou, China). M_n_ is the number-average molecular weight, M_w_ is the weight-average molecular weight. PDI is the polydispersity index.

### 2.2. Preparation of Sample

PVDF, PMMA and PLLA were dried in a vacuum oven overnight at 80 °C in order to remove moisture before processing. The blend of PVDF/PMMA/PLLA/SZ-01 (*w*/*w*/*w*/*w*, 30/30/40/0.05, the total amount of sample preparation is 50 g) was prepared in a Haake Polylab QC mixer (Thermo Fisher Scientific, Waltham, MA, USA) with a rotation speed of 20 rpm for 3 min and then 50 rpm for 10 min at 200 °C. PVDF/PMMA/PLLA/SZ-01 films were hot-pressed at 200 °C and 10 MPa for 10 min. The films were melted for 30 min to obtain round PVDF/PMMA mixed-phase at 200 °C and then isothermal crystallized at 145 °C. A Soxhlet extraction apparatus containing chloroform was used to selectively etch PMMA and PLLA. After that, the PVDF microspheres were obtained by a freeze-drying method. More details can be found in our previous work [23]. PLLA films were hot-pressed at 200 °C and 10 MPa for 13 min. A rectangular PLLA film with the size of 2 cm × 3 cm was taken and several drops of chloroform were dropped to make it swell. The PVDF spheres were quickly coated on the swollen film to prepare our specimens. Uniaxial tension was performed on PLLA films along direction A in a water-bath at 70 °C to certain draw ratios (1.2, 1.3, 1.5). Biaxial tension was performed stepwisely. In other words, the specimen was stretched along one direction to a certain draw ratio, followed by deformation in the other direction to the same draw ratio. 

### 2.3. Microstructure Characterization

The sample was soaked in liquid nitrogen for 5 min and cut in the middle with two pairs of tweezers. The morphologies of composite film were observed by scanning electron microscope (SEM, Hitachi S-4800, Tokyo, Japan) with an accelerating voltage of 5.0 kV. The fraction of the exposed area (this parameter can be defined as the area which has not been covered by porous PVDF spheres) on the composite film was carried out by ImageJ (Wayne rasband National Institutes of Health, Stapleton, USA, software version: ImageJ 1.51j8). Relevant data were obtained through the fraction of the exposed area on the composite film in SEM images.

### 2.4. Drop Shape Analysis (DSA)

We used a rectangular PLLA film with the size of 2 cm × 3 cm to measure the CAs and SAs. To test sample wettability, static contact angles (CAs) and sliding angles (SAs) were measured by drop shape analysis (DSA, DSA-100, Krüss). The volume of droplets used for the contact angle measurement is 3 μL, at a room temperature of 25 °C and humidity of 21%. Five DSA tests were performed for each sample. The average value of them has been adopted.

### 2.5. Dynamic Mechanical Analysis (DMA)

Dynamic mechanical analysis (DMA) was carried out using DMA Q-800 (TA Instrument, New Castle, PA, USA), and the specimens were tested with a heating rate of 3 °C/min from 30 to 120 °C with a fixed amplitude of 5 μm and frequency of 5 Hz.

## 3. Results and Discussion

**Figure 1:**
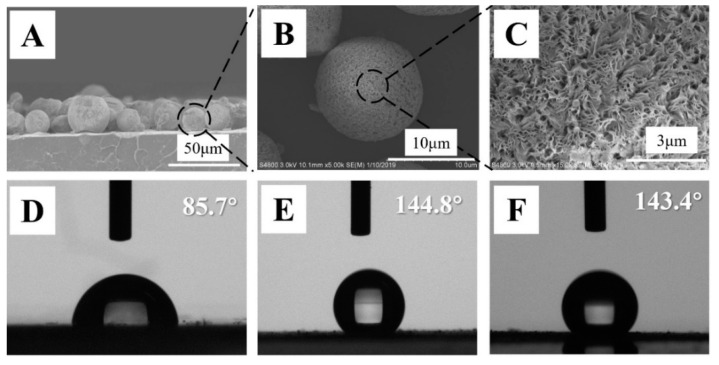
SEM images of PVDF@PLLA composite fracture surface (**A**), porous PVDF (**B**,**C**). (**D**–**F**) shows the water contact angles (CAs) on neat PLLA film (**D**), porous PVDF spheres @tapes (**E**) and PVDF@PLLA composite film (**F**).

Porous PVDF spheres were prepared according to the strategy discussed in our previous work [23]. In short, in a ternary blend of PVDF/PMMA/PLLA with compatibilizers (SZ-01, 0.5 wt%), phase separation produced PVDF/PMMA mixed phase and PLLA phase. The latter acted as a matrix due to the higher volume fraction. Upon etching with chloroform, both PLLA matrix and PMMA in mixed phase were removed, yielding porous PVDF spheres with a diameter of several microns (Figure 1B). In the fabrication of porous PVDF spheres, therefore, PMMA acts as the agency for narrow nanopores. On these spheres, there are narrow pores in nanometers (Figure 1C) that resulted from the exclusion behaviors of PMMA during the crystallization of PVDF (i.e., narrow pores from the crystallization template) [24,25,26]. The PLLA film with a thickness of 300 μm was prepared by means of hot-pressing. Several drops of chloroform (a good solvent for PLLA, poor solvent for PVDF) were produced on PLLA film to swell the surface layer. Then, porous PVDF spheres were placed and pressed slightly on the swollen PLLA film. In this way, the porous spheres were embedded partly and fixed on the surface of PLLA film (Figure 1A). The free (un-embedded) spheres were removed by blowing with compressed nitrogen. To assess the water wettability on PLLA films and PVDF spheres, drop shape analysis (DSA) measurements have been performed. The results are shown in Figure 1D–F. On neat PLLA films, the water contact angle is roughly 85.7°, suggesting a hydrophilic surface. To measure the contact angle of porous PVDF spheres, multi-layers of them have been prepared on transparent tape. In this case, water droplets come in contact with only PVDF spheres, which is an efficient way to avoid the influence of the supporting layer on the contact angle. As shown in Figure 1E, the angle reaches 144.8°. This result makes it clear that porous PVDF spheres are quasi-superhydrophobic. Figure 1F illustrates the water contact angle on porous PVDF spheres supported by PLLA films, i.e., PVDF@PLLA. It exhibits a magnitude of 143.4°, which was similar to that of the porous spheres (Figure 1E) but much higher than neat PLLA (Figure 1D). The high WCA of porous PVDF spheres can be interpreted as follows: On one hand, PVDF itself is a well-known hydrophobic material, which has been widely used in water treatment [27,28,29]. On the other hand, there is hierarchical roughness on the surface of porous PVDF spheres. The roughness in the former can be calculated according to the method shown in Figure S2. In microns, roughness comes from the neighboring spheres (Figure 1A,B) while there are porous structures in nanometers on each sphere (Figure 1C). The combination of hydrophobic property and hierarchical roughness contributes to the quasi-superhydrophobicity. This is very similar to the well-known lotus leaves effect, in which there are numerous waxy bumps in microns and nanometers [13,16,17]. In Figure 1F, the composite PVDF@PLLA exhibits a similar contact angle with Figure 1E. This result indicates that most of the PLLA film surface has been covered with porous PVDF spheres. It is difficult for water droplets to come into contact with PLLA, which is the reason for the resultant air-pockets and the consequent Cassie state [30].

**Figure 2:**
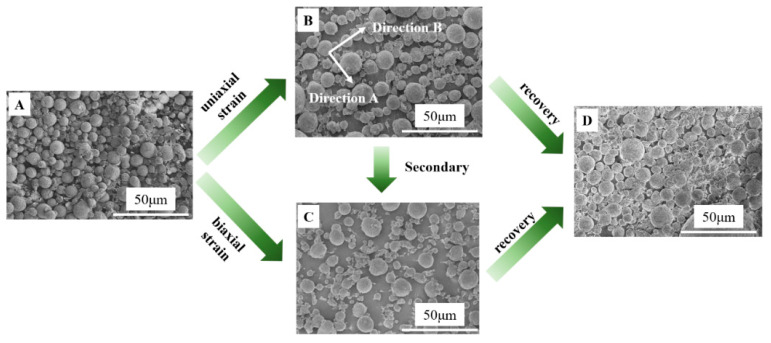
SEM images of PVDF@PLLA composite surface with various deformations. (**A**) Original, (**B**) 50% uniaxial strain, (**C**) 50% biaxial strain, (**D**) recovery.

PLLA is a typical shape memory polymer, in which the amorphous matrix and tiny crystals of it play the role of the shape recovery phase and shape fixed phase, respectively. In this work, the crystallinity of PLLA is 3.6% (determined by DSC, data not shown here). The shape memory effect endows PLLA with the ability to be deformed at high temperatures and be fixed by cooling down, which has been investigated in detail in our previous work [31,32,33]. The glass transition temperature of PLLA (63.4 °C, determined by means of dynamical mechanical analysis, Figure S3) acts as the switching temperature of the shape memory effect. The surface morphology of the obtained composite film was observed by means of SEM. In Figure 2, only typical SEM images have been shown since they play key roles in the transition between adhesive and anti-adhesive performances. After preparation, the whole surface of PLLA film has been covered by porous PVDF spheres (Figure 2A), accounting for the high value of the water contact angle shown in Figure 1F. This is the permanent shape of the composite film. In a 65 °C water bath (above the switching temperature of PLLA), the composite film was stretched to the draw ratio of 1.5 in a certain direction (defined as direction A) to obtain a temporary shape followed by cooling down to room temperature to fix it. To show the difference of distance among neighboring spheres in direction A and direction B, the software of Nano Measurer has been employed. The distance in direction A increases remarkably (>4 μm, Figure 2B) while it is still comparable with that before deformation in the other direction (called direction B). The composite film goes from isotropic to anisotropic upon uniaxial tension. After that, two clamps were used to fix the edge of the composite film in direction A. Then, a secondary tension process was carried out along the edge in the other direction (direction B). This is so-called biaxial tension. Upon biaxial tension with a draw ratio of 1.5 in direction A and direction B, the distance of the neighboring spheres in two directions is comparable (Figure 2C). That is to say, the composite film changes back to an isotropic state again. Of course, it is also facile to perform biaxial tension in another way, i.e., the tension in two directions simultaneously (from Figure 2A to Figure 2C directly). During uniaxial and biaxial tensions, porous PVDF spheres embedded on the PLLA film surface remain still while PLLA can be deformed. In this process, the covered area (defined as the area covered by porous PVDF spheres) remains almost constant. At the same time, the whole area of PLLA exhibits a much higher magnitude, leading to the higher area fraction of the exposed PLLA area (defined as the area which has not been covered by porous PVDF spheres, Figure 2B,C). The quantitative analysis will be discussed in the following sections. Upon heating at 65 °C, the composite film recovers to its permanent shape in a relatively short period (Video S1 and Figure S2) from either a uniaxial tension state (Figure 2B) or biaxial tension state (Figure 2C). In Figure 2D, almost all the surface of composite PVDF@PLLA film has been covered by porous PVDF spheres again, which resembles so closely with that before deformation (Figure 2A).

**Figure 3:**
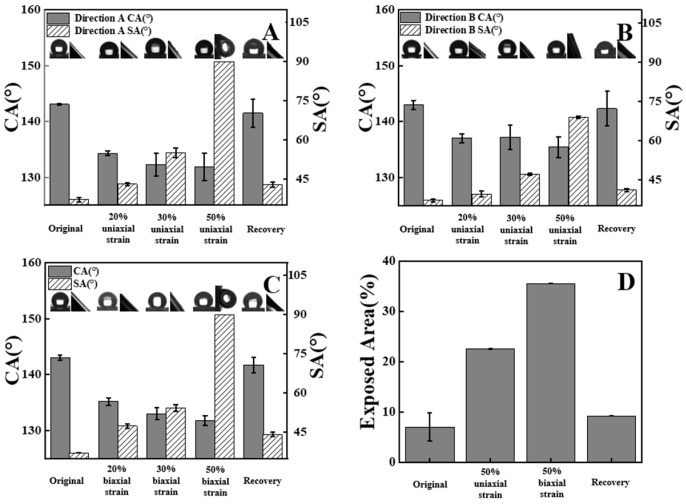
Water contact angles (CAs) and sliding angles (SAs) of the composite films as a function of draw ratios observed from direction A (**A**), direction B (**B**), during uniaxial tension and biaxial tension (**C**). (**D**) Shows the fraction of the exposed area on the composite film.

Upon uniaxial tension, the distances of PVDF porous spheres in two directions are different, suggesting the anisotropic composite film. Therefore, a series of uniaxial tensions with various draw ratios were carried out. Figure 3A,B shows the contact and sliding angles during uniaxial tension. The original film surface exhibits a high contact angle (143°) and low sliding angle (37°, Video S2), corresponding to the enhanced mobility, anti-adhesive performance and lotus leaves effect. In direction A (Figure 3A), the contact angle decreases from 143° to 132° while the sliding angle increases from 37° to 90° (Video S3) upon uniaxial tension (Figure 3A). Here, 90° means that the droplets cannot roll down even when the specimen was placed vertically. The lower and higher magnitudes of contact angles and sliding angles indicate the adhesive state and rose petals effect. When the specimen is put into a water bath (65 °C), it recovers to the initial state upon thermal stimuli. Both contact angle and sliding angle are comparable with those in an as-prepared specimen. A similar thing happens in direction B. However, uniaxial tension produces higher sliding angles in direction A. The sliding angle difference between direction A and direction B increases with the increasing draw ratio. It is 5° and reaches 21° in the case of a draw ratio of 1.2 and 1.5, respectively. These results confirm that composite film exhibits anisotropic wettability after uniaxial tension. Biaxial tension yields isotropic wettability in two directions (Figure 3C). The contact angle decreases to 135°, 133°, 132° with a draw ratio of 1.2, 1.3 and 1.5, respectively. At the same time, the sliding angles increase monotonously. Obviously, it changes from an anti-adhesive state (lotus leaves effect) to an adhesive state (rose petals effect). After recovery, the film surface goes back to the lotus leaves effect (i.e., low sliding angle). According to the discussion above, the original and deformed films exhibit the lotus leaves effect and rose petals effect, respectively. The transition between them can be repeated.

The reversible transition shown in Figure 3 can be attributed to the exposed area fractions among porous PVDF spheres which are under the control of deformation and recovery of shape memory PLLA films. At the very beginning, the whole surface of PLLA films has been covered by PVDF spheres. The surface of the composite film exhibits the quasi-superhydrophobicity resulting from the hydrophobic property of PVDF and hierarchical roughness from neighboring spheres and narrow pores on each sphere (Figure 1B,C). When water droplets contact the surface of composite films, they slip easily along the surface since there are numerous air pockets among spheres. The whole surface resembles lotus leaves so closely, on which there are hierarchical waxy dumps. This is the reason for the anti-adhesive state shown in Figure 3. Upon uniaxial or biaxial tension, porous PVDF spheres remain still while the supporting PLLA film has been deformed. As a result, more and more area has been exposed to water droplets (Figure 2B,C). The exposed area of the composite film surface was statistically analyzed using the Image J software. As shown in Figure 3D, the exposed area fraction increases remarkably (from 7% to 36%) after stretching. The exposed PLLA film surface can be regarded as the hydrophilic defects since PLLA itself exhibits a water contact angle of 85.7° (Figure 1D). On the composite film (PVDF@PLLA) surface, therefore, the increase of area fraction of the hydrophilic part contributes to the slightly lower contact angle and enhanced adhesive behaviors (higher sliding angles) shown in Figure 3A–C. During the rolling-down of water droplets, it is the exposed PLLA surface (hydrophilic part) that prevents the continuous mobility of them. When the specimen is heated above the switching temperature of shape memory PLLA, the supporting film recovers to its permanent shape, resulting in the complete coverage of its surface by porous PVDF spheres again (<10% exposed area, Figure 3D). It is difficult for water droplets to contact PLLA film due to the Cassie state on PVDF. In this case, the wettability and adhesion of composite film were determined by porous PVDF spheres. Then, the surface of the composite film switches back to the lotus leaves effect (Figure 3A–C). During the transition discussed above, the Wenzel and Cassie states play key roles in determining the contact angle as well as sliding angles. The distance between hydrophobic PVDF spheres depends crucially on deformation. When the area of hydrophilic defect (i.e., exposed PLLA) is small, the repulsive effect of porous PVDF spheres is so strong that it is impossible for water droplets to come in contact with PLLA, corresponding to a Cassie state. In the case of a higher area fraction of hydrophilic defect, the water droplet can contact PLLA directly, producing a Wenzel state. In this work, therefore, the composite surface exhibit a mixed state of Cassie and Wenzel states because of the different diameters of PVDF spheres ranging from 2 to 15 μm and the hydrophilic defects with different exposed areas.

**Figure 4:**
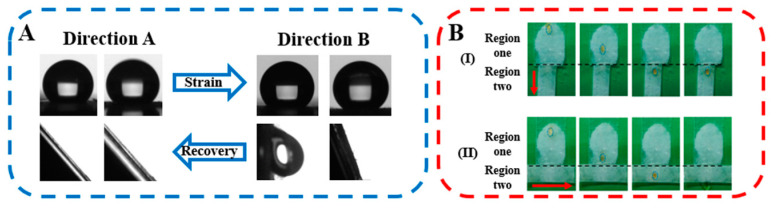
(**A**) Contact (up) and sliding (down) angles in two directions on the surface before (left) and after (right) uniaxial tension (DR = 1.5) and the controlled droplet transportation based on the anisotropic wetting behaviors on the PVDF@PLLA surface. (**B**) The red arrow illustrates the direction of uniaxial tension.

To show the anisotropic wettability clearly, our attention has been paid to the composite film before and after uniaxial tension (Figure 4). On the as-prepared specimen, both contact angles and sliding angles in two directions are similar (143° and 37° respectively, Figure 3A). Upon uniaxial tension with the draw ratio of 1.5, contact angles in direction A and direction B exhibit different magnitudes (132° and 135°, respectively, Figure 3A and Figure 4A). The sliding angles increase remarkably and are different in two directions. In direction B, it is 68° while the water droplet cannot move even when the specimen is placed vertically in direction A (i.e., 90°). Based on the difference in sliding angles in the two directions, the PVDF@PLLA composite film surface can be used for controlled droplet transportation. For this purpose, a specimen containing two regions has been prepared (Figure 4B). In region one, the surface of the composite film is in the original (or recovered) state while it is stretched along the red arrow direction in region two. To achieve the droplet movement on the attained surface, the specimen was placed in two cases at a tilted angle of 70° (Videos S4 and S5). This value is higher than 68° but lower than 90°. In case I, the water droplet exhibits mobility driven by gravity in region one since the original state corresponds to the lotus leaves effect. In region two, it is hard for it to move due to the enhanced adhesive performance resulting from the exposed hydrophilic defects of PLLA (Figure 2B) and the resultant rose petals effect (Figure 3). Consequently, the droplet has been captured. In case II, however, the droplet can pass the whole surface including two regions easily, which can be attributed to the lower sliding angles and lotus leaves effect (Figure 3).

## 4. Conclusions

In this work, the programmable transition between adhesive/anti-adhesive performances has been achieved on the surface of composite PVDF@PLLA film (i.e., porous PVDF spheres on shape memory PLLA). After preparation, the whole surface of the composite film is covered by porous PVDF spheres. The combination of hydrophobic PVDF and hierarchical roughness from neighboring spheres and narrow pores on each sphere endows the surface with a high contact angle and low sliding angle, corresponding to a Cassie state, anti-adhesive performance and lotus leaves effect. The shape memory effect makes it facile to deform PLLA film at a high temperature by means of uniaxial or biaxial tension. The higher distance among porous PVDF spheres resulting from enlarged PLLA film produces some exposed hydrophilic defects (i.e., PLLA) which can prevent the continuous mobility of water droplets. This is the reason for the Wenzel state, adhesive performance and rose petals effect. After thermal stimuli, the supporting PLLA film recovers to its initial state. In this case, the surface of the composite film is covered by porous PVDF spheres again, resulting in the lotus leaves effect. The composite film works successfully in controlled droplet transportation. Our results provide an efficient solution for the programmable and switchable transition between adhesive and anti-adhesive performances on (quasi-)superhydrophobic surfaces.

## Data Availability

Not applicable.

## Supplementary Materials

The following are available online at https://www.mdpi.com/article/10.3390/polym14030374/s1, Figure S1: Structural formula of P(MMA-co-GMA) (A) and PLLA (B); Figure S2: The profile of a specimen surface (red dash line). The black line has been defined as the height of zero; Figure S3: Changes of tan δ with increasing temperature for PLLA; Figure S4: Images of stretched and recovered PVDF@PLLA surfaces. (A) Draw ratio = 1.5, (B) recovered; Video S1: The process of shape recovery; Video S2: The sliding angle of the original film; Video S3: The sliding angle in the direction A after 50% stretching; Video S4: Dynamic video of Case Ⅰ; Video S5: Dynamic video of Case Ⅱ.
