# Supplementary material for: Programmable Transition between Adhesive/Anti-Adhesive Performances on Porous PVDF Spheres Supported by Shape Memory PLLA
Source: Polymers (Basel). 2022 Jan 19;14(3):374. doi: 10.3390/polym14030374 (PMC8839783; doi:10.3390/polym14030374)
Supplement: Supplementary file 1 [file polymers-14-00374-s001.zip › polymers-1495763-supplementary.pdf]

Supplementary Material for

Programmable Transition between Adhesive/Anti-adhesive Performances on Porous PVDF Spheres Supported by Shape Memory PLLA

Jiaqin Zhao, Liang Zhang, Xiong Cheng, Jiayao Wang, Yongjin Li and Jichun You*

College of Material, Chemistry and Chemical Engineering, Hangzhou Normal University, Hangzhou 311121, China; Jiaqin_Zhao@163.com (J.Z.); liangzhang1128@gmail.com (L.Z.); 2019112009005@hznu.stu.edu.cn (X.C.); jiayao-wang@outlook.com (J.W.); yongjin_li@hznu.edu.cn (Y.L.)

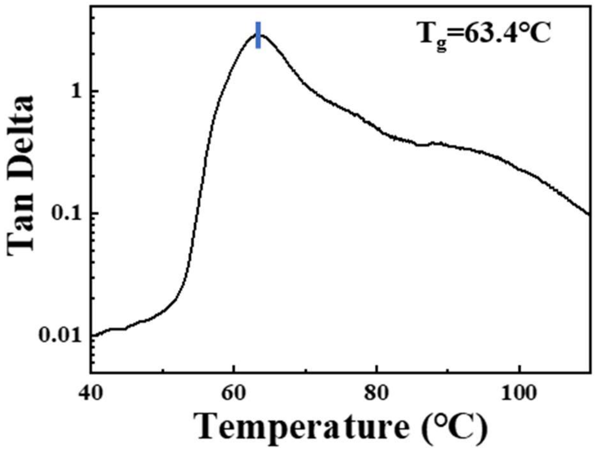

Figure S1. Changes of tanδ with increasing temperature for PLLA.

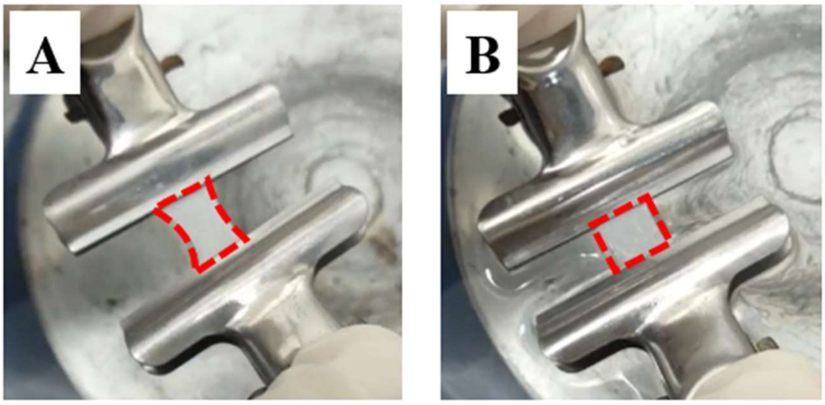

Figure S2. Images of stretched and recovered PVDF@PLLA surfaces. (A) draw ratio = 1.5, (B) Recovered.
